# O035. Headaches in Mitochondrial Disorders

**DOI:** 10.1186/1129-2377-16-S1-A68

**Published:** 2015-09-28

**Authors:** Catello Vollono, Giudo Primiano, Anna Losurdo, Serenella Servidei, Giacomo Della Marca

**Affiliations:** Unit of Neurophysiopathology and Sleep Medicine, Department of Geriatrics, Neurosciences and Orthopedics, Catholic University, Rome, Italy; Istituto Auxologico Italiano, Department Neurology, S. Luca Hospital, Milan, Italy

## Background

Headaches are a well known feature of Mitochondrial Disorders (MCDs). However, no systematic epidemiological data are available in large populations of patients.

We aimed to describe the prevalence and the characteristics of headache in a large group of patients with mitochondrial encephalomyopathies.

## Methods

We studied all consecutive patients referred to our Neuromuscular Unit, during a 6-month period. Ninety-three patients (aged 15 to 78 years, 31 males) with a typical phenotype of MCDs, underwent a structured diagnostic headache interview, using an operational diagnostic tool following the IHS criteria. If they met the criteria for primary headache, they were included in the “Headache Group” (HEAD+). The other patients were collected in the “No Headache Group” (HEAD-). Clinical, neuroradiological, and neurophysiological data were compared between groups. Mann-Whitney U-test was used to analyze numeric variables; Fisher's exact test was used to analyze nominal variables. Binary logistic regression analysis was performed to identify risk factors of headache.

## Results

Headaches were reported in 35.48% of patients. Migraine was the most common headache. The patients of the Headache Group were younger (HEAD+ = 45.5±17.2 years; HEAD- = 54.5±14.8 years; U-test = 7.393; p = 0.007), increased prevalence of epilepsy (p = 0.0103), myoclonus (p = 0.0309), stroke (p = 0.0290), EEG focal slow abnormalities (p = 0.0359), EEG epileptic focal abnormalities (p = 0.0425), and decreased prevalence of muscle weakness (p < 0.0001) and EEG normal pattern (p = 0.0136). Multivariate analysis showed that HEAD+ was significantly associated with absence of muscle weakness (CI = 1.007-14.894; p = 0.049) and EEG abnormalities (CI = 1.347-78.085; p = 0.025).

## Conclusions

Migraine has a higher prevalence in MCDs compared to population-based data. Our findings are consistent with the widely hypothesized role of mitochondria in the migraine pathophysiology.

Written informed consent to publication was obtained from the patient(s).

